# Primary Care Clinician and Clinic Director Experiences of Professional Bias, Harassment, and Discrimination in an Underserved Agricultural Region of California

**DOI:** 10.1001/jamanetworkopen.2019.13535

**Published:** 2019-10-23

**Authors:** Michelle Ko, Armin Dorri

**Affiliations:** 1 Division of Health Policy and Management, Department of Public Health Sciences, University of California, Davis

## Abstract

**Importance:**

Many rural and agricultural communities experience hardship from a shortage of clinicians. The aging of the clinician population threatens future supply in these areas. Developing policies to build a sustainable workforce requires the understanding of experiences from those currently in medical practice. Previous research about rural clinicians has primarily sampled non-Latinx white men, and to a lesser extent, non-Latinx white women; to date, no study has examined differences by race/ethnicity, sexual orientation, or gender identity.

**Objective:**

To describe the professional experiences of a diverse group of primary care clinicians and clinic directors in an underserved rural and agricultural region of California.

**Design, Setting, and Participants:**

In this qualitative study, semistructured in-depth qualitative interviews were performed with clinicians and clinic directors from December 1, 2017, to December 31, 2018, with a present or recent medical practice in the central San Joaquin Valley region of California. Participants (N = 26) consisted of physicians, nurse practitioners, and clinic directors practicing in primary care settings. Settings included solo and group private practice, academic training programs, community health centers, and rural health clinics.

**Main Outcomes and Measures:**

Personal experiences as primary care clinicians and clinic directors, and perceived associations with gender, race/ethnicity, sexual orientation, and gender identity.

**Results:**

Of 26 primary care clinicians and clinic directors interviewed, 16 (62%) identified as female, 12 (46%) identified as non-Latinx white, and 3 (12%) identified as a member of a sexual and gender minority group. Participants who self-identified as female, nonwhite, and of certain sexual orientation and gender identity minority groups described burnout from bias, harassment, and hostility in their professional relationships with colleagues and health care staff. These experiences intensified their feelings of community isolation and professional isolation. Harassment and institutional discrimination were factors in the decision of participants to change practices or exit the region entirely. Discriminatory acts against members of sexual and gender minority groups were the most severe, including threats to licensure and denial of hospital admitting privileges. In contrast to the minority group participants, the remaining participants expressed little to no awareness of these negative experiences, or the association between these experiences and retention.

**Conclusions and Relevance:**

Professional harassment and discrimination may hamper efforts to improve clinician and clinic director recruitment and retention in underserved rural and agricultural areas and may pose barriers to addressing health disparities within those communities. Additional investigation appears to be needed to assess the extent to which professional harassment and discrimination affect clinicians and clinic directors in similar communities across the United States.

## Key Points

**Question:**

Are the experiences of a diverse group of primary care clinicians and clinic directors in an underserved rural and agricultural area in California associated with professional satisfaction and retention?

**Findings:**

In this qualitative study of 26 primary care clinicians and clinic directors, diverse by gender, race/ethnicity, and/or sexual orientation and gender identity, their responses to interview questions described bias, harassment, and discrimination from colleagues, staff, and local health systems. Participants noted burnout, isolation, changing medical practices, or leaving the region entirely from these experiences.

**Meaning:**

The findings suggest that professional harassment and discrimination may exacerbate existing clinician shortages in rural and agricultural areas, further amplifying geographic disparities in health care access and quality.

## Teaser

This qualitative study describes the professional experiences and the perceived associations with gender, race/ethnicity, sexual orientation, and gender identity of a diverse group of primary care clinicians and clinic directors in an underserved rural and agricultural region of California.

## Introduction

Underserved rural and agricultural areas have long struggled to recruit and retain health care clinicians. In the next decade, geographic difficulties in access to care may worsen as an estimated 40% of physicians proceed toward retirement.^1^

To address clinician shortages, it is critical to understand the experiences of those presently in medical practice. Previous research^2,3,4,5^ had focused on the clinician’s affinity for rural culture, community integration, and professional networks. However, most studies^2,3,4,5^ examined the experiences of male physicians. The handful of studies^6,7,8,9,10^ about female physicians in rural medical practice additionally emphasized community integration of partners and families, and colleague support for work flexibility and maternity leave. Though the proportion of physicians who are female has steadily increased during the previous 2 decades, women remain the minority of physicians practicing in primary care specialties.^11^ Almost no study^2,3,4,5^ to date has examined rural clinicians of nonwhite race/ethnicity or differing sexual orientation or gender identities. Greater diversity of perspectives is needed, as a substantial body of research not specific to rural areas has found that minority groups experience professional isolation, harassment, and discrimination throughout training and practice.^12,13,14,15,16,17,18,19,20,21,22,23,24,25,26,27,28,29,30,31,32,33,34,35^

The aim of our study was to examine the practice experiences of a diverse group of primary care clinicians and clinic directors in a highly underserved rural and agricultural region. We conducted in-depth interviews of clinicians and clinic directors from California’s Central San Joaquin Valley (hereafter referred to as the Valley). The Valley is a global leader in agricultural production yet consistently ranks at the bottom of the state for supply of primary care clinicians.^36^ Of the region’s primary care physicians, 28% are female and 28% are underrepresented racial/ethnic minority groups (defined as African American/black, American Indian/Alaska Native, Hispanic/Latinx [defined as a gender-neutral word for people of Latin-American descent], Native Hawaiian/Pacific Islander, or Southeast Asian); the proportion who identify as sexual and gender minority (SGM) group is unknown.^36^

## Methods

### Study Design

Semistructured in-depth interviews were conducted using a grounded theory approach, involving simultaneous and iterative data collection and analysis, a focus on actions and processes, and construction of theory.^37^ In recognition that researchers approach investigations with a priori knowledge and assumptions, the constructivist perspective was adopted that results are not independent theories, but rather constructed interpretations formed from both participant and researcher views and experiences. All participants were informed of study procedures and granted verbal consent to participate after description of the study procedures was stated by one of us (M.K.). The protocol and subsequent modifications to the interview guide and sampling strategy were approved by the University of California, Davis Institutional Review Board. The Standards for Reporting Qualitative Research (SRQR) guideline was used to prepare the report of the findings.

### Context and Sampling Strategy

The Valley spans 8 counties that generate approximately $33 billion in agricultural revenues per year.^38^ The terms *rural* and *agricultural* were used to describe the context; in addition to rural areas, the Valley contains towns, cities, and urban centers. The predominance of agriculture reinforces a strong rural identity throughout the region,^39^ and socioeconomic conditions are consistent with those of underserved rural areas. Valley counties lead the state in both agricultural employment^40^ and poverty.^41^ Of the 4 million residents in the region, 44.5% are uninsured or supported by Medicaid^41^ and 51.4% of the residents are of Latinx ethnicity (Non-Latinx white [34.1%], Asian American [6.6%], and African American/black [4.5%]). An estimated 5.4% of residents identify as a member of a sexual and gender identity minority (SGM) group.^41^

A snowball sampling approach was used, drawing from professional networks, supplemented with letters, paper advertisements, and social media. The initial aim was to recruit participants across a range of practice settings, genders, and race/ethnicity. In accordance with grounded theory, theoretical sampling was engaged with modification of recruitment procedures as new categories emerged.^37^ This included specific efforts to recruit SGM group participants via participant referrals, professional networks, and the Gay and Lesbian Medical Association provider directory, a list of health care professionals identifying as lesbian, gay, bisexual, transgender, and queer (LGBTQ) friendly.^42^ Each clinician and clinic director was contacted at least twice by mail, telephone, and email (for a minimum of 6 attempts). Because participants also recommended colleagues who exited the region because of negative professional experiences, those participants who had left the Valley were included. Considerable challenges in recruiting participants included in the SGM group were also encountered, and efforts were also sought to contact those with previous experience practicing in the Valley, if not in present medical practice in the region.

### Data Collection

One of us (M.K.) conducted interviews via telephone and in-person from December 1, 2017, through December 31, 2018. All participants provided verbal consent to participate. The interviewer emphasized that participants could withdraw from the study or decline to answer specific items at any time, and that responses would be published anonymously, including removal of potential practice or regional identifiers. Interviews took place from 45 to 90 minutes and they were digitally audio recorded and transcribed. Participants received a $50 gift card and completed an online survey about self-identified gender, race/ethnicity, and practice characteristics. Transcript and survey data were labeled with anonymized alphanumeric study identifiers.

The initial interview guide contained open-ended questions about practice challenges, strategies, and personal journeys to practice in the Valley. The first 3 interviews elicited differences by self-identified gender sexual orientation, and thus the guide was modified to include probes about how these identities were associated with practice experiences. The Box lists questions from the interview guide related to the themes presented in this article, and the eAppendix in the Supplement presents a complete interview guide. Participants were asked to reflect about perceived differences for colleagues.

Box. **Initial and Modified Interview Questions Regarding Clinician and Clinic Director Experiences**

**Initial Interview Questions**

- What do you see as your biggest challenges in providing timely, high quality, primary care in your community?
- Can you talk about your journey to medicine, from your childhood to your undergraduate education, and then your medical training?

**Probes Added to Initial Interview Guide**

- Do you feel as if your gender has affected your professional experiences in any way?
- Do you feel as if your race or ethnicity has affected your professional experiences in any way?
- Do you feel as if your sexual orientation or gender identity has affected your professional experiences in any way?

Each question was also repeated in reference to the participants’ colleagues, eg, How about your colleagues? Do you feel as if gender has affected their professional experiences in any way?

### Analysis

Two reviewers (M.K. and S. Kumari, BA) used an immersion and crystallization approach to individually conduct line-by-line coding and identify emerging categories, followed by review and consensus. Two reviewers (M.K. and A.D.) then used constant comparative methods for codes and themes within and between interviews. Consistent with a grounded theory approach, coding emphasized processes and examined not only what was said, but how participants responded to queries and what was not said.^39^ The online qualitative analysis platform Dedoose (SocioCultural Research Consultants LLC) was used to organize transcripts and interviewer notes and to conduct coding and memo-writing.

One reviewer (M.K.) identified as cis-female heteronormative, East-Asian American and has extended family who have lived in the Valley for 3 generations. The other reviewer (S. Kumari, BA) identified as cis-female heteronormative South-Asian American from a high-poverty, medically underserved, nonrural community. Another reviewer (A.D.) identified as a gay cis-male, Middle-Eastern American individual.

From initial data collection to completion of analysis, one of us (M.K.) presented the design and findings to students, trainees, and researchers who met 1 or more of the following characteristics: (1) currently living in the Valley; (2) self-identified origins from the Valley; (3) interested in rural and/or agricultural health; and (4) experienced in providing primary care in the Valley. These presentations provided an opportunity to triangulate the study approach, findings, and interpretation with alternative perspectives.

## Results

Of 26 primary care clinicians and clinic directors who were interviewed, 16 (62%) identified as female, 12 (46%) identified as non-Latinx white, 3 (12%) as Asian, and 3 (12%) identified as a sexual and gender minority group (Table 1). Three participants had left the Valley: 1 participant left 4 years before the interview and 2 participants left during the previous year. To protect participant confidentiality, results are presented with limited descriptors, eg, gender pronouns will be used only in the discussion of gender but not in the discussion of race/ethnicity. Themes and illustrative quotations are presented in Table 2.

**Table 1.** Characteristics of Participating Primary Care Clinicians and Clinic Directors^a^

Characteristic	Participants (N = 26)
	No. (%)
Gender^b^	
Male	10 (38)
Female	16 (62)
Race/ethnicity^b^	
African American	1 (4)
Asian	3 (12)
Latinx	5 (19)
Non-Latinx white	12 (46)
Other	2 (8)
Missing	1 (4)
Practice type^c^	
Solo or small group (1-9 clinicians)	9 (35)
Large group or academic (≥10 clinicians)	9 (35)
Community health center or rural health clinic	11 (42)
Specialty	
Family medicine	15 (58)
Pediatrics	3 (12)
Internal medicine	5 (19)
Other^d^	3 (12)

^a^ Seven participants did not complete the online survey. When possible, missing information was derived from interview data and publicly available practice information, (eg, the practice website).

^b^ Gender and race/ethnicity are self-identified.

^c^ Total number exceeds 100% because participants reported practicing at multiple settings.

^d^ Other included obstetrics and gynecology (n = 2) and psychiatry (n = 1).

**Table 2.** Principal Themes From Primary Care Clinician and Clinic Director Interviews

Theme	Quotation
Bias, harassment, and hostility	Misogynistic—that was the culture [in our clinic].
	I have kids and a family, so I was always on the blacklist.
	It’s not my personal mission to make everybody not racist—it took too much of my energy.
	I was off every committee. I would raise my hand and it was like I wasn’t even there.
	“A patient said, ’My doctor told me you were gay.’ Like what does that have to do with anything? It’s kind of like, ‘I’m going to send you to a doctor but he’s black.’”
Community and professional isolation	The only ones who are open—and it’s only recently—they’re in ER, anesthesia, some setting where you don’t have patients refusing to come see you. Primary care providers are terrified of what [being supportive of SGM patients] would do to their practice.
Hostile environments and institutional discrimination	I felt unheard and unappreciated and disrespected and so I left.
	[Our practice] had already been run out of the [hospital] system. They made it super clear they didn’t want us here.
Lack of recognition of biases	Why wouldn’t I sign up saying I’m open to seeing Christian patients?
	[Some] called the county medical society to tell them how horrible it was, that it’s just wrong, because physicians shouldn’t know anything about LGBT health or be friendly to LGBT patients.

### Bias, Harassment, and Hostility

Many participants reported experiences of bias, harassment, and discrimination based on gender, race/ethnicity, and/or sexual orientation and gender identity. Participants emphasized that negative interactions occurred with colleagues, staff, and administrators, but did not occur with patients.

Twelve of the 16 female participants expressed frustration after receiving negative comments regarding family obligations, resistance to scheduling flexibility, and general lack of respect. Three participants reported harassing behaviors from male colleagues (including sexually inappropriate jokes and degrading comments about females) and the use of medical practice and hospital computers to view pornographic material. One clinician noted difficulty obtaining specialty consultations from male physicians, and another female clinician described how male physicians in the community frequently dismissed her practice as “the girl group.” Several participants perceived gender-associated barriers to attaining leadership positions, either within their own organizations or within the region (Table 2).

Three participants described feeling fatigue from colleagues’ racial biases. The African American/black participant reported persistent racial microaggressions from other physicians, staff, and patients, including being told that they did not look like a real doctor, and noting that colleagues were quick to perceive them as angry. One of the white participants expressed anger regarding staff racist comments about patients with Medicaid coverage (Table 2).

Two SGM participants described overt hostility, including receiving expletive-laden notes and vandalism of their cars. One participant was asked to step down from leadership of hospital boards and medical organizations after their SGM status became known (Table 2).

One SGM participant reported no experiences of professional harassment associated with sexual orientation or gender identity. However, in contrast with the other 2 participants, who identified specific changes in behaviors after coming out or being outed, the third participant expressed uncertainty whether colleagues or other staff knew their SGM status, using phrases such as they “thought” others knew.

### Community and Professional Isolation

Multiple participants described how professional isolation from their minority group status exacerbated their sense of geographic isolation. One female participant described the regional medical society as an “old boys club.” The African American/black participant worried that the lack of diversity in the local community would create a negative environment for their children and cited this as the primary reason for leaving the Valley.

The 3 SGM participants reported nearly all of their SGM colleagues had left the Valley, and those who remained kept their status closely guarded. They characterized local residency programs as more tolerant, but that SGM trainees left the region after completion of their training because of stigma in the broader community. One non-SGM participant who identified as an SGM advocate noted that hospital-based health care professionals might come out, but none in primary care (Table 2).

### Hostile Environments and Institutional Discrimination

Four female participants reported that they changed medical practices as a result of gendered harassment and discrimination. A fifth female participant described a similar work environment that led her to leave her previous position, though she did not attribute her experiences to her gender (Table 2).

Two SGM participants described institutional practices taken against them and their SGM colleagues. In one case, hospital staff filed complaints to hospital administrators as well as to the state medical licensing board. The participant became embroiled in a year-long investigation because staff cited that the participant’s out status created a hostile work environment. Participants noted that several hospitals denied admitting privileges if a clinician’s SGM status became known. In another case, the sole hospital in the community retracted the clinician’s admitting privileges, and thus they were “run out of town” because they were unable to secure insurance contracts. Participants cited the cumulative emotional and financial toll of anti-SGM harassment and discrimination as motivating their decisions to leave the Valley (Table 2).

### Lack of Recognition of Biases 

In contrast to minority group participants, the remaining participants (whether by gender, race/ethnicity, or sexual orientation and gender identity) described limited or no association of these identities with practice experiences, burnout, job change, or leaving the region.

When asked about gender differences, male clinicians primarily noted patient preferences for female clinicians. Two participants noted female clinicians’ increased family obligations. Whereas female clinicians described such challenges in association with practice structure and interactions with colleagues, male clinicians characterized these issues as the consequence of their female colleagues’ personal choices for family life.

The greatest difference arose in discussions about sexual orientation and gender identity, where most participants generally evaded direct responses. Participants either restated their response to the gender question, or they used single-phrase denials. Two non-SGM participants described colleagues as “biologically male,” and misgendered the pronouns he/him, when discussing transgender females, possibly suggesting their own resistance to gender nonconforming identities. These responses contrasted sharply with the discussions about gender and race/ethnicity; participants provided expansive responses, even when the participant concluded no or limited differences.

Only 2 participants related negative professional experiences among their SGM colleagues. One described encountering resistance when attempting to encourage colleagues to identify their medical office as an LGBTQ-friendly practice. They reported that colleagues privately expressed support for SGM populations, but the colleagues resisted signaling public support (Table 2).

### Recent Changes in Professional Climate

Female and Latinx participants suggested that work environments were improving with demographic changes among physicians and the community. Two women described changing policies and introducing education within their medical practices to counter gender bias. Among our 4 female participants who reported no negative experiences associated with gender, all participants had been practicing for fewer than 5 years in the region, and 2 had joined practices consisting of mostly female clinicians.

Participants were less sanguine about the future outlook for SGM health care professionals in the region. One described assisting SGM health care professionals to leave the Valley, characterizing the process as an extraction. Two participants suggested that the movement for SGM rights at the state level had produced a cultural backlash in the region, with 1 participant perceiving that medical practice exits had increased in recent years. Another participant expressed concern that expansion or consolidation of health systems with ethical or religious directives would compel more SGM practicing primary care to leave.

## Discussion

To our knowledge, this is the first study to describe how experiences of bias and discrimination may have contributed to health care professional turnover in a predominantly rural and agricultural region. Previous studies^6,8,9^ of rural female physicians did not elicit these findings, but did identify issues of work inflexibility and lack of supportive professional relationships.^6,8,9^ Unlike previous studies,^6,8,9^ we asked questions specific to gender and conducted interviews during a period of national dialogue about female harassment, thus participants may have felt encouraged to discuss these types of experiences.^43^ Our findings were consistent with multiple studies about sexism in health care,^32,33,44,45,46,47^ which have also documented poor mental health and leaving medical practice as consequences.^48^ In highly underserved communities, such experiences carry substantial implications for health care access and quality for residents.

To date, few studies have addressed the experiences of racial/ethnic minority groups practicing medicine in rural and/or agricultural areas.^49^ In 1 study from 1996,^49^ minority group physicians participating in rural National Health Service Corps sites felt less accepted by their local medical communities, and a few cited community racism and bigotry as sources of dissatisfaction.^49^ Studies not specific to rural or agricultural areas have found minority group physicians, medical students, trainees, and faculty^14,15,30,50,51,52^ experienced professional isolation and barriers to advancement in medicine.

Our findings regarding SGM participants appeared to be consistent with the existing literature on nonrural clinicians and clinic directors that has found pervasive harassment, and discrimination in professional settings.^22,24,26,27,34,53,54,55,56,57^ Some reported changing jobs because of a hostile professional climate.^58^ Trainees have expressed fear of practicing medicine outside of urban areas if the community was not supportive of their sexual orientation^33^; 27% of LGBTQ health care professionals have reported witnessing acts of SGM group discrimination against coworkers.^54^ Eliason et al^53^ found that acts of explicit bias against LGBTQ health care professionals have declined, but our findings suggest that explicit and implicit bias, particularly enacted at administrative and institutional levels, may have an association with resulting behavior and decisions made by SGM health care professionals.

Our study is notable for eliciting perspectives from participants who exhibited symbolic and aversive views and behaviors. In symbolic behaviors, members of social majority groups perceive disadvantages faced by minority groups as self-inflicted and criticize minority group individuals for excessively demanding recognition.^59^ This perception was reflected among the participants who questioned the need for attention to LGBTQ-related health issues. With aversive behaviors, members of majority groups outwardly express sympathy yet may still engage in discriminatory practices.^60^ This was evident among the clinicians who expressed concern for LGBTQ patients but who declined to identify as LGBTQ friendly. Both patterns were consistent with a larger context of discrimination; both shed light on barriers to addressing discrimination if health care professionals are unaware of or unable to acknowledge such practices.

Our findings may differ from previous studies because the proportion of participants practicing in group settings was higher than that of rural areas on average.^2,3,4,5^ The potential for professional harassment and discrimination may increase when clinicians are working within organizations, rather than in solo practice. However, such working arrangements appear to be important to examine given increasing consolidation of health systems,^61^ including in rural areas.^62^ Community health centers are likewise growing as organizations because of their substantial expansion under the Affordable Care Act.

### Implications

Biases, harassment, and discrimination are not unique to rural or agricultural communities or clinicians and clinic directors—a growing body of literature illustrates how these experiences are pervasive throughout health care, from students and trainees to community and academic settings. However, we believe our findings raise concerns for highly underserved areas in which health care professionals and patients have fewer options to mitigate the associations. Underrepresented minority group medical students and clinicians report higher levels of stress, anxiety, and depression, all of which can be escalated among SGM clinicians who conceal their identities.^58,63^ These conditions accelerate clinician burnout, which is associated with the provision of lower quality of care and greater job turnover. We cannot quantify the extent to which negative professional experiences contribute to clinician exit from rural communities, but we believe our findings suggest that these considerations require greater scrutiny in the context of severe clinician shortages.

We also identified ways other actors in the health care system can drive clinicians out of rural communities. Because physicians are barred from direct employment by hospitals in California, it is unclear whether those who encounter discrimination from hospital staff or administration (or other entities, such as health insurance plans) can claim violations of Title VII civil rights protections^64^ from employment discrimination. The US Supreme Court will begin to review 3 cases this fall that address whether Title VII entails protections by sexual orientation or gender identity.^65^ The US Department of Health and Human Services has also signaled greater support for health care entities to abstain from medical practices for which they have religious or moral objections.^66^ Without federal protections, increased diversity among clinicians is critical to reducing discrimination experienced by patients in the SGM group. The US Department of Health and Human Services has proposed removing regulations that interpreted Title IX to include protections against health care discrimination based on sexual orientation or gender identity.^67^

The lack of support for minority group health care providers in underserved rural and/or agricultural areas may block progress on addressing health disparities. In 2011, the Institute of Medicine called for greater understanding on health care inequities for SGM populations,^68^ and the American Medical Association and the Association of American Medical Colleges have called for increasing education on the health needs of SGM populations.^69,70^ A recent study on internal medicine residents found no difference in SGM knowledge in urban areas with high SGM group prevalence vs other locations,^71^ and a survey specifically of rural clinicians found large variation in clinician knowledge on SGM health.^72^ Education is critical for reducing disparities but also requires a clinician population that is supportive of these initiatives, and our findings suggest the limits to this approach with a predominantly non-SGM clinician workforce.

### Limitations

Because our study was conducted in 1 region of 1 state, the findings may not be generalizable to other regions and clinicians. The Valley contains metropolitan areas, but the predominance of agriculture and clinician shortages are more consistent with rural areas. Second, we may have recruited participants who were more likely to have similar and/or negative experiences. We also acknowledge that there may be differences between those individuals presently in practice in the Valley and those who have left the region; however, we note that themes were consistent irrespective of location. The aim of our study was not to characterize a general trend but to identify and develop potential emerging theories. Thus, the focus was on data collection to encompass a variety of participant experiences and views.^39^ Third, despite repeated efforts through different modalities, we were unable to recruit additional SGM participants. We thus refrained from concluding that we have achieved data saturation for SGM perspectives. Given the severity of the consequences described by our participants, it was not surprising that health care professionals would be reluctant to participate, and more research on this population appears to be needed. In addition, we note also that we did not recruit participants in tribal settings and/or those employed by Indian Health Services; given the distinct cultural contextual and service differences, we concluded that experiences in these settings merit separate study.

## Conclusions

We believe our findings highlight the importance of the diversity and inclusion of health professions in underserved rural areas. We conducted an exploratory study to understand the experiences of a diverse group of primary care clinicians; our findings suggest that more investigation is needed to determine the extent to which bias, harassment, and discrimination may be occurring in other rural areas in the country and to determine their association with primary care access and quality.

## Notes

Supplement. **eAppendix.** Interview Guide
