# Supplementary material for: Primary Care Clinician and Clinic Director Experiences of Professional Bias, Harassment, and Discrimination in an Underserved Agricultural Region of California
Source: JAMA Netw Open. 2019 Oct 23;2(10):e1913535. doi: 10.1001/jamanetworkopen.2019.13535 (PMC6820031; doi:10.1001/jamanetworkopen.2019.13535)
Supplement: Supplement. — eAppendix. Interview Guide [file jamanetwopen-2-e1913535-s001.pdf]

Supplementary Online Content

Ko M, Dorri A. Primary care clinician and clinic director experiences of professional bias, harassment, and discrimination in an underserved agricultural region of California. *JAMA Netw Open*. 2019;2(10):e1913535. doi:10.1001/jamanetworkopen.2019.13535

eAppendix. Interview Guide

This supplementary material has been provided by the authors to give readers additional information about their work.

© 2019 Ko M et al. *JAMA Network Open.*

eAppendix. Interview Guide

Thank you for taking the time to speak with us. The purpose of our study is to get current perspectives on the challenges faced by healthcare providers in the San Joaquin Valley, and to learn about successful strategies that providers are engaged in to meet those challenges.

As a reminder, your participation in this study is voluntary- you may ask to withdraw at any time. Also, although we will be asking questions about your practice, you are not expected to, and should not, provide any information that would be considered protected health information of your patients.

1. What do you see as your biggest challenges in providing timely, high quality, primary care in your community?

2. What strategies have you used to address those challenges? Which approaches have you found to be most effective, and why?

3. *(If not already addressed by participant)* In addition to the challenges we have already discussed, there are several relatively newer conditions in the San Joaquin Valley that can also present new difficulties, or make existing challenges more difficult. To what extent are you experiencing any changes due to:
   a. Federal efforts in immigration enforcement
   b. Uncertainty in healthcare policy
   c. Drought and access to water, or other environmental concerns

4. For these challenges, what strategies have you employed, or think you will employ?

5. One challenge that has seemed particularly difficult to fix is the chronic shortage of healthcare providers. Given your role as someone who chose to practice in the Valley, we would like to hear more about your own experiences. Can you talk about your journey to medicine, from your childhood, to your under graduate education, and then your medical training?

6. *(If not already discussed)* How did you end up coming to work here? What do you think influenced you to practice in the Central Valley?

© 2019 Ko M et al. *JAMA Network Open.*

Additional questions

Added after 3 interviews, to expand on emerging categories

7. *(If not already discussed)*
   a. Do you feel as if your gender has affected your professional experiences in any way?
   b. Do you feel as if your race or ethnicity has affected your professional experiences in any way?
   c. Do you feel as if your sexual orientation or gender identity has affected your professional experiences in any way?
   d. *(As an extension to questions a-c)* How about your colleagues?

Added after 16 interviews, in response to request from funder

8. Can you describe your experiences in caring for farm/agricultural workers?

© 2019 Ko M et al. *JAMA Network Open.*
